# Research hotspots and frontiers of alcohol and epilepsy: A bibliometric analysis

**DOI:** 10.1002/npr2.12421

**Published:** 2024-03-01

**Authors:** Wenhui Liu, Huan Li, Simei Lin

**Affiliations:** ^1^ Department of Neurology, Institute of Neuroscience, Key Laboratory of Neurogenetics and Channelopathies of Guangdong Province and the Ministry of Education of China The Second Affiliated Hospital of Guangzhou Medical University Guangzhou China

**Keywords:** alcohol, bibliometric analysis, CiteSpace, epilepsy, VOSviewer, Web of Science

## Abstract

**Purpose:**

Alcohol is implicated in epileptogenesis and seizures attack. An increasing number of studies about alcohol and epilepsy have been published. We aimed to assess research trends and hot spots in the field of alcohol and epilepsy.

**Patients and Methods:**

Literature concerning alcohol and epilepsy was systemically searched through the Web of Science database. Collaborative maps were quantitatively analyzed by using the VOSviewer and CiteSpace tools.

**Results:**

A total of 1578 papers about the field of alcohol and epilepsy were taken into analysis, which was written by 6840 authors from 2153 institutions in 85 countries, published in 676 journals, and cited 79 667 references from 10 750 journals. The United States was the leading country and had close ties with others. The University of Toronto was the most productive institution. *Alcoholism‐clinical and experimental research* was the fastest‐growing journal. Richard J. Bodnar was the author contributing the most literature. Analysis of keywords showed epilepsy, alcohol, seizures, alcohol withdrawal, and management were common themes.

**Conclusion:**

The presented study conducted the first bibliometric analysis of the field of alcohol and epilepsy, which will provide insights into the latest progress, evolution paths, frontier research hot spots, and future research trends in the field.

## Abstract 2

In this study, we conducted the first bibliometric analysis in the field of alcohol and epilepsy, which explored the hot spots and frontiers over the past 30 years and generated the corresponding knowledge maps using CiteSpace and VOSviewer.

## Graphical abstract

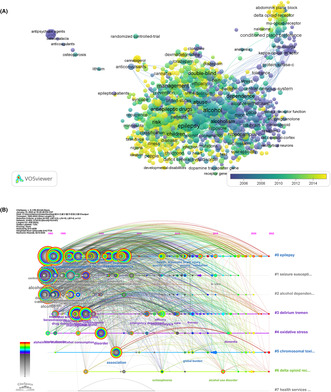

## 1 INTRODUCTION

Epilepsy is a chronic brain disease characterized by recurrent, paroxysmal, and transient central nervous system dysfunction caused by abnormal excessive discharge of neurons.^1^, ^2^, ^3^, ^4^ Previous research indicated that alcohol consumption is a risk factor for epilepsy.^5^, ^6^, ^7^, ^8^, ^9^, ^10^ The International League Against Epilepsy (ILAE) proposed alcohol intake as one of the risk factors for epilepsy and advised patients to refrain from drinking.^9^, ^11^, ^12^ The role of alcohol in epileptogenesis and seizures attack also attribute great attention from scientific researchers. The field of alcohol and epilepsy is booming, with increasing output. However, the papers in this field have not been quantitatively analyzed by a bibliometrics approach, which will provide an overview and help in exploring the research frontiers from the quantified perspective.

In this study, we conducted the first bibliometric analysis in the field of alcohol and epilepsy, which explored the hot spots and frontiers over the past 30 years and generated the corresponding knowledge maps. This study provided insights into the latest progress, evolution paths, frontier research hot spots, and future research trends in the field.

## 2 MATERIALS AND METHODS

### 2.1 Data source and retrieval strategy

This study retrieves all available articles until December 31, 2022, from the Web of Science database (SCI‐EXPANDED & SSCI), which covers the oldest publications with firm coverage. The search formula is “ALL = ((epilepsy OR seizure) AND (liquor OR alcohol))”. Duplicate results were manually removed.

### 2.2 Data analysis and visualization

Through scientific mapping procedures, researchers can visually analyze the structure, dynamic patterns, and trends of the field, which can help in identifying the evolution path, classical literature, and the frontier of the discipline.^13^ Bibliometric maps were constructed by the CiteSpace^14^, ^15^, ^16^ and VOSviewer,^17^, ^18^, ^19^ including countries, institutions, published journals, co‐cited journals, authors, co‐cited authors, references, co‐cited references, and keywords.^20^, ^21^, ^22^, ^23^ The parameter setting of CiteSpace was Method (LLR), Time Splicing (1993–2022), Text Processing (all term source), and Node types (choose the node we need). We determined the parameter settings for the VOSviewer according to the data type, data source, and the selected analysis and counting methods. The Centrality reflects the value of nodes in the networks (Centrality >0.1, indicating having an important influence).

## 3 RESULTS

### 3.1 The scientific outcome in the field of alcohol and epilepsy

A total of 1578 papers were retrieved for analysis. These papers were written by 6840 authors from 2135 institutions in 85 countries, were published in 676 journals, and cited 79 667 references from 10750 journals.

### 3.2 Analysis of publication years

**FIGURE 1:**
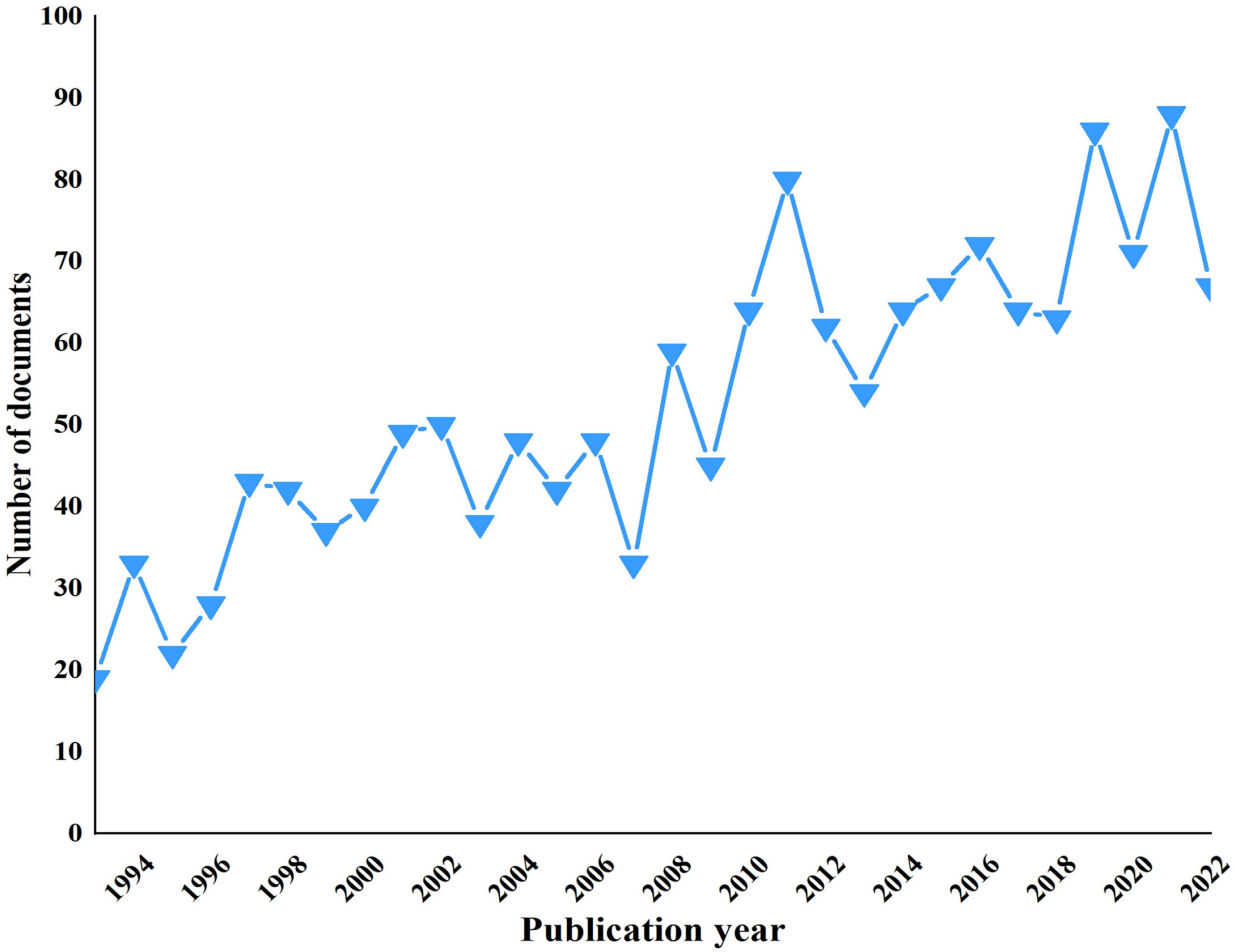
Schematic illustration of analysis of publication years.

The annual publications were on an increased trend, especially after 2007 (Figure 1). The number of annual publications increased from 19 in 1993 to 67 in 2022 with a peak of 88 papers in 2021.

### 3.3 Analysis of countries

**FIGURE 2:**
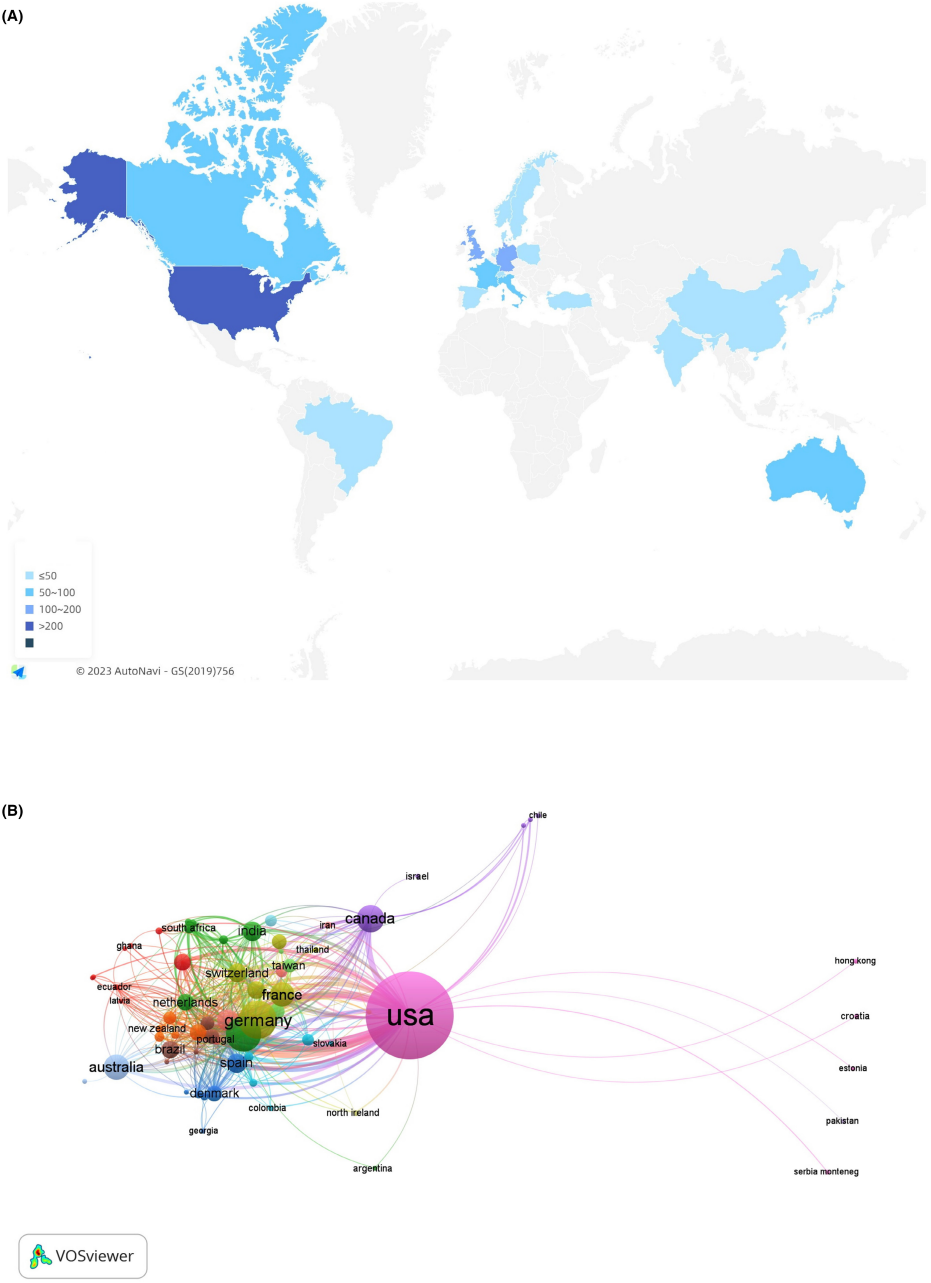
Schematic illustration of analysis of countries. (A) Top 20 countries productivity in included literature; (B) Country collaboration map, where circles denote countries, and lines denote their collaborations.

The contributed authors were from 85 countries (Figure 2A). The USA (609, Centrality = 0.5) was the most frequent country. Germany (142, Centrality = 0.16), Britain (124, Centrality = 0.28), Canada (80, Centrality = 0.05), and Australia (71, Centrality = 0.05) were the remaining countries in the top five (Table 1). Figure 2B showed the close cooperative relations among countries by the VOSviewer tool. The USA shared highly close ties with Germany, Australia, France, and other countries.

**TABLE 1:** The top 10 productive countries and institutions.

Rank	Countries	Count	Centrality	Rank	Institutions	Count	Centrality
1	USA	609	0.5	1	University of Toronto	21	0.03
2	Germany	142	0.16	2	Oregon Health and Science University	20	0.01
3	Britain	124	0.28	3	Columbia University in the City of New York	15	0.02
4	Canada	80	0.05	4	Boston University	15	0.03
5	Australia	71	0.05	5	CUNY Queens College	14	0.00
6	France	68	0.08	6	Mayo Clinic	13	0.01
7	Italy	55	0.16	7	University of Erlangen‐Nuremberg	12	0.00
8	Spain	45	0.03	8	King's College London	12	0.02
9	India	44	0.02	9	University of California, San Diego	11	0.01
10	Switzerland	41	0.03	10	Harvard University	10	0.00

### 3.4 Analysis of institutions

**FIGURE 3:**
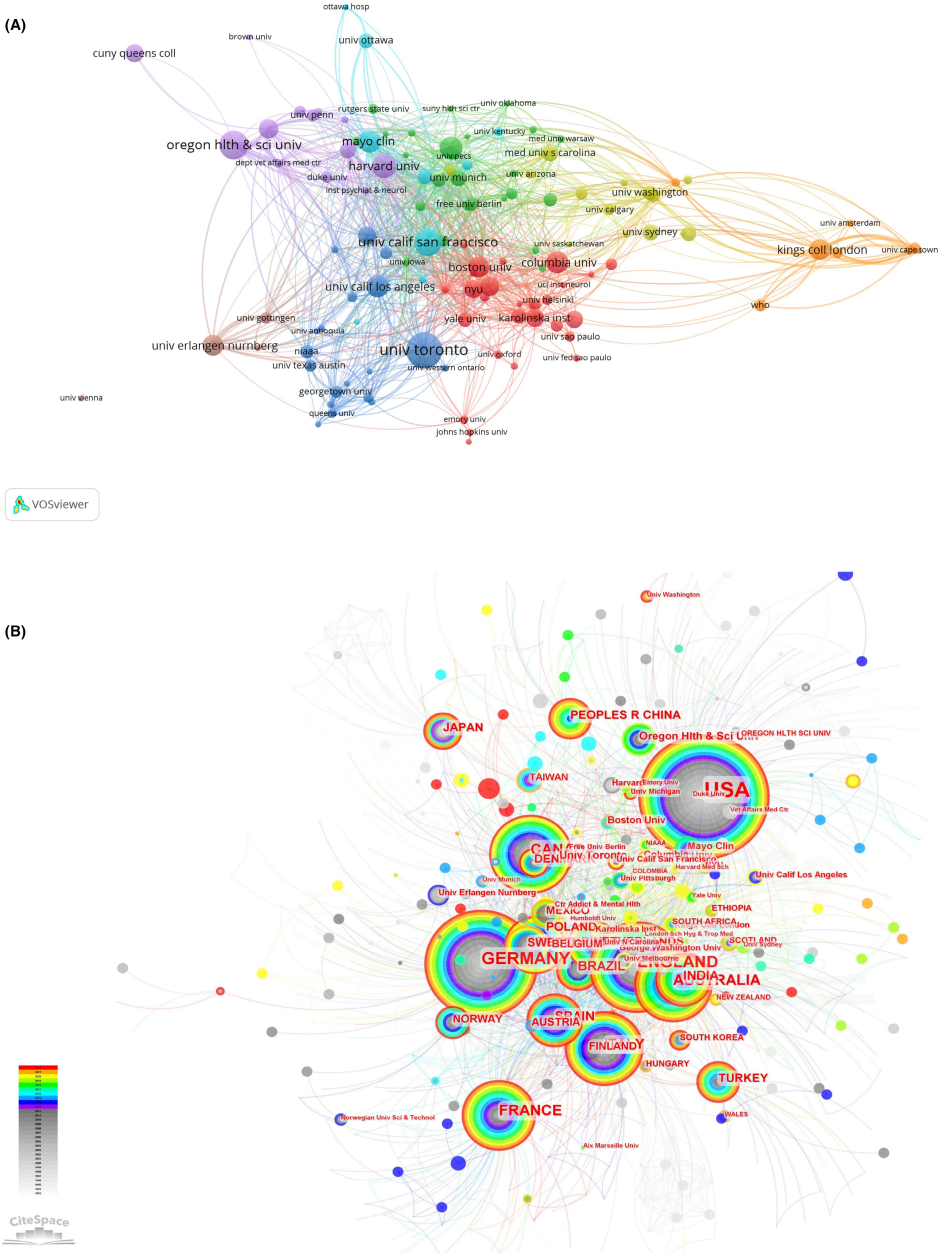
Schematic illustration of analysis of institutions. (A) Institution collaboration map; (B) Network visualization of countries and institutions, which circles represent the institutions and the countries to which belong, the size of the circles represents the number of published papers, the colors of the circles represent the different years of publications, and the connecting lines represent the cooperation between institutions.

The top 10 institutions contributed 143 publications (9.06%) (Table 1). The top three were the University of Toronto (21, Centrality = 0.03), Oregon Health and Science University (20, Centrality = 0.01), and Columbia University in the City of New York (15, Centrality = 0.02). Seven of the top 10 institutions were from the United States, with the remaining three from Canada, Germany, and the Britain, respectively. The collaboration between institutions was shown in Figure 3A. The University of Toronto and Boston University had the closest cooperation with other institutions. To fully understand the collaborative relationships between countries and institutions, we visualized their collaboration by the CiteSpace tool. The USA was the home to most institutions and articles (Figure 3B). These findings showed that the USA and its institutions may play critical roles in the field of alcohol and epilepsy.

### 3.5 Analysis of journals

The 1578 papers taken into analysis in this study were published in 676 journals. Table 2 listed the top 10 high‐output journals. The journal with the highest impact factor (IF) was *Neurology*. The journal with the most published was *Alcoholism‐clinical and experimental research* (*n* = 66), followed by *Epilepsia* (*n* = 42) and *Epilepsy & behavior* (*n* = 41). Among these journals, five of the top 10 were neurology‐related, three were alcohol‐related, and the remaining two journals were Pharmacy‐related and Multidisciplinary sciences‐related, respectively.

**TABLE 2:** The top 10 most productive journals published.

Rank	Journal	Count	JCR	IF (2021)
1	Alcoholism‐Clinical and Experimental Research	66	Q3	3.928
2	Epilepsia	42	Q1	6.74
3	Epilepsy & Behavior	41	Q3	3.337
4	Alcohol and Alcoholism	40	Q3	3.913
5	Seizure‐European Journal of Epilepsy	31	Q3	3.414
6	Peptides	24	Q2	3.867
7	Alcohol	21	Q3	2.558
8	Neurology	18	Q1	12.258
9	Acta Neurologica Scandinavica	15	Q2	3.915
10	PLoS One	15	Q2	3.752

Abbreviations: IF, Impact Factor; JCR, Journal Citation Reports.

### 3.6 Analysis of co‐cited journals

**FIGURE 4:**
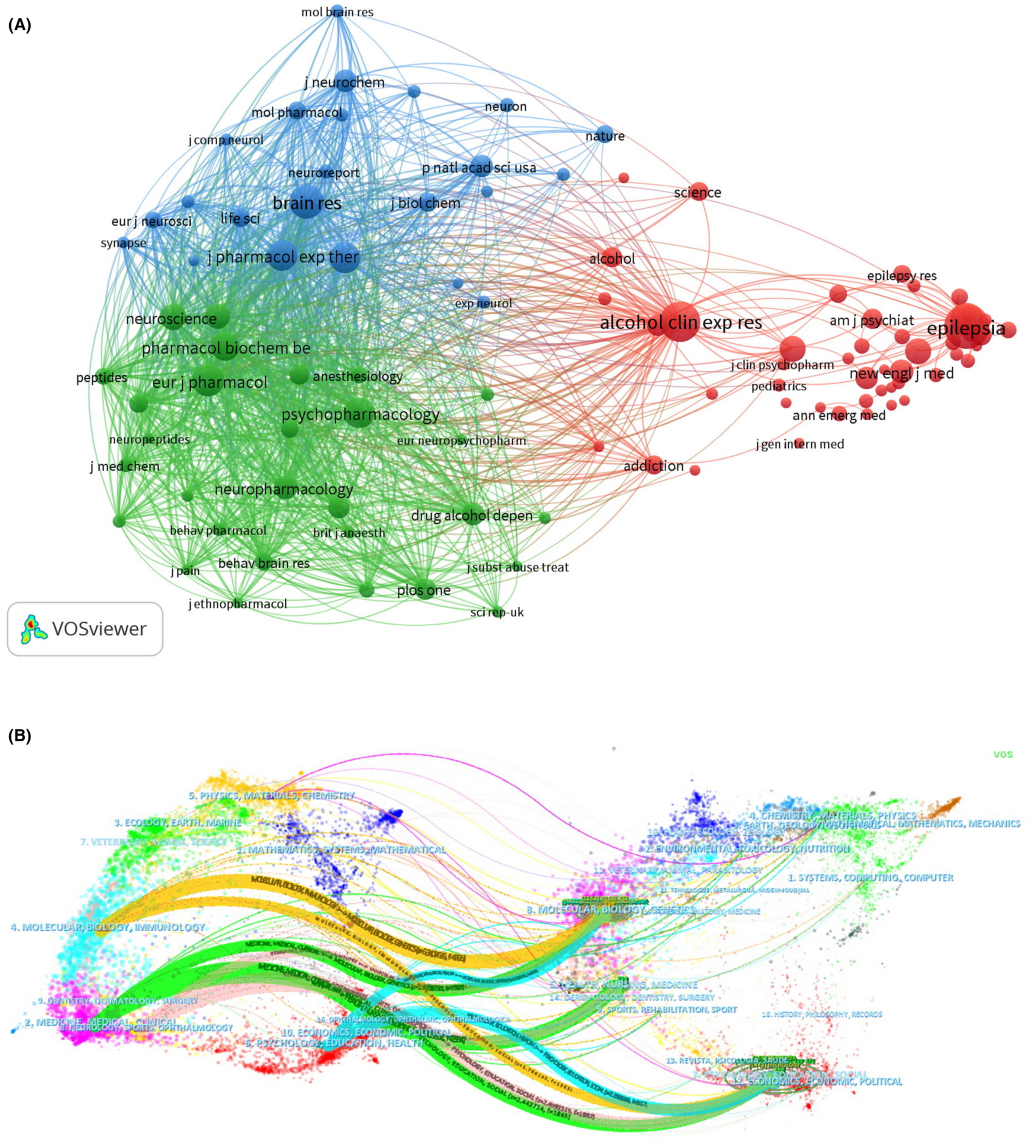
Schematic illustration of analysis of co‐cited journals. (A) The co‐citation journal visualization network can be divided into three different clusters (red clustering represented the journal of clinical, green represented the journal of pharmacology, and blue was for that of neurosciences); (B) The dual‐map overlay and corresponding disciplines, which left side of the map shows journals, and the right side shows cited journals. Different colored paths show the flow and connection of knowledge from different research fields.

Co‐citation is the frequency with that two articles are cited together.^24^ By establishing the structure of the pattern of linkages among papers, the co‐citation provides a tool for monitoring the development of scientific fields and assessing the degree of the interrelationship among specialties.^24^ The more frequently the journals are co‐cited, the closer their academic relationship.^24^, ^25^ The top 10 co‐cited journals are shown in Table 3, among which Epilepsia had the most frequencies of co‐citations (2639 times). The top 100 journals, being co‐cited by at least 144 times, were used for the analysis through the VOSviewer tool. The network can be divided into three main clusters, including clinical medicine, pharmacology, and neurosciences (Figure 4A).

**TABLE 3:** Top 10 journals for co‐citation.

Rank	Journal	Citations	JCR	IF (2021)
1	Epilepsia	2639	Q1	6.74
2	Alcoholism‐Clinical and Experimental Research	2105	Q3	3.928
3	Brain Research	1564	Q3	3.61
4	Neurology	1445	Q1	12.258
5	European Journal of Pharmacology	1286	Q2	5.195
6	Journal of Pharmacology and Experimental Therapeutics	1280	Q2	4.404
7	Journal of Neuroscience	1279	Q1	6.709
8	Psychopharmacology	1226	Q2	4.415
9	Pharmacology Biochemistry and Behavior	1015	Q3	3.697
10	Neuroscience	889	Q3	3.708

Figure 4B illustrated the topic distribution of the journals through the dual map overlay. We found three main citation paths. These three paths indicated that studies published in Molecular, Health, and Psychology were often cited by those published in Immunology, Dentistry, and Medicine.

### 3.7 Analysis of authors

**FIGURE 5:**
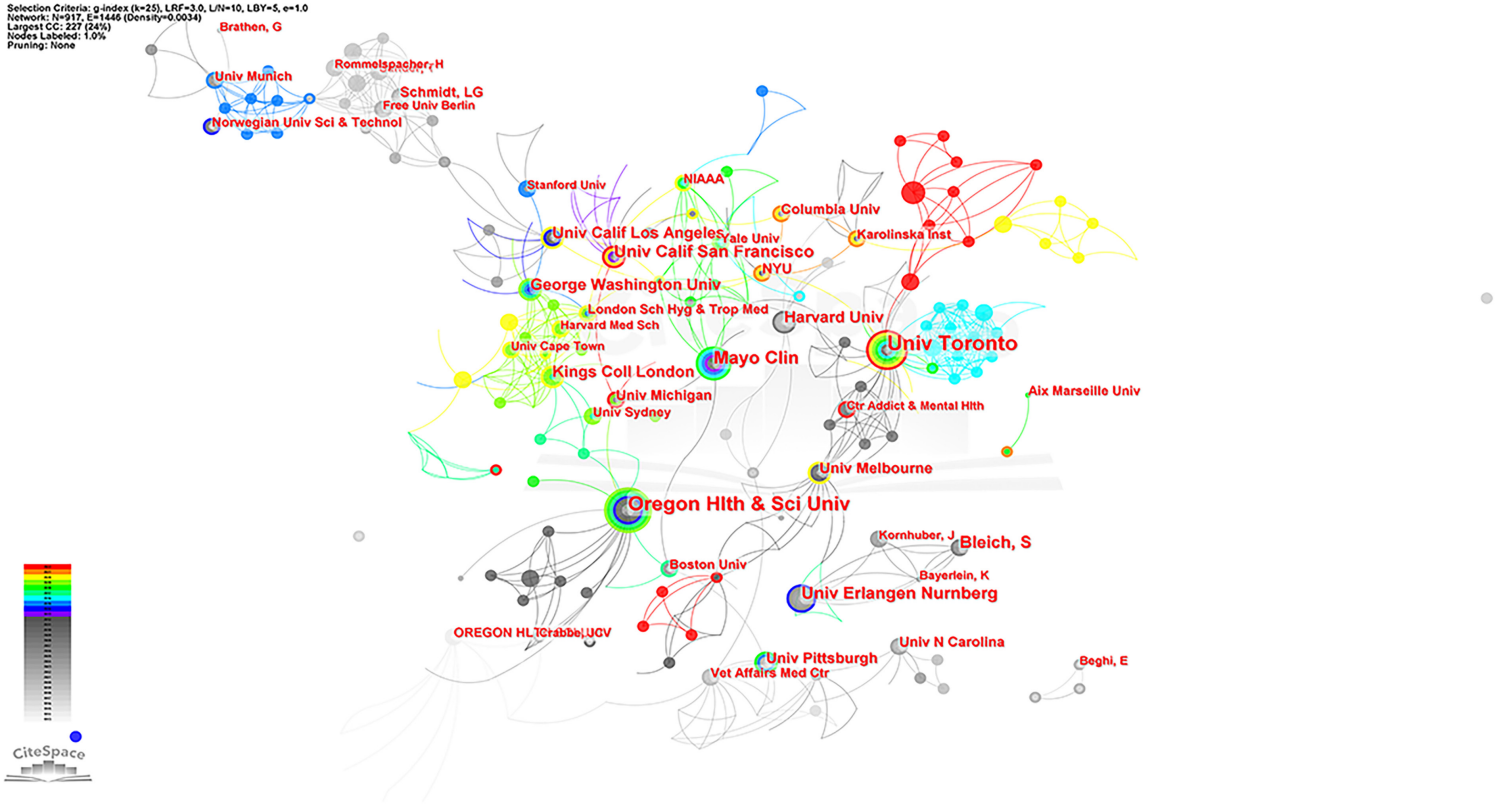
Network visualization of authors and institutions, which circles represent the authors or institutions, the circle sizes represent the number of publications, and the links represent the relationship between the authors and the institutions.

The number of published papers represents the author contributions and research activities in a field.^26^ Table 4 showed the top 10 authors with the most published papers. Richard J. Bodnar ranked first (*n* = 16, 2.34%), followed by Lutz G. Schmidt (*n* = 11, 1.61%) and Sara N. Bleich (10, 1.46%). We used CiteSpace to visualize the authors and institutions included in the literature. The University of Toronto has the most authors in this field (Figure 5). Uncoincidentally, Richard J. Bodnar also has the closest cooperation with others.

**TABLE 4:** The top 10 productive authors.

Rank	Author	Count (%)	Co‐cited author	Citation
1	Richard J. Bodnar	16 (2.34)	W. Allen Hauser	87
2	Lutz G. Schmidta	11 (1.61)	Marc A. Schuckit	86
3	Sara N. Bleich	10 (1.46)	John T. Sullivan	63
4	Johannes Kornhuber	10 (1.46)	Howard C. Becker	62
5	John C. Crabbe	10 (1.46)	Michael F. Mayo‐Smith	61
6	Prosper N'Gouemo	10 (1.46)	Matheus B. Victor	58
7	Tomas Sander	9 (1.31)	Matti Hillbom	55
8	Hans Rommelspacher	8 (1.17)	Michael E. Brown	54
9	Jerzy Samochowiec	7 (1.02)	James C. Ballenger	53
10	Richard W. Olsen	7 (1.02)	Robert S. Fisher	50

### 3.8 Analysis of co‐cited authors

**FIGURE 6:**
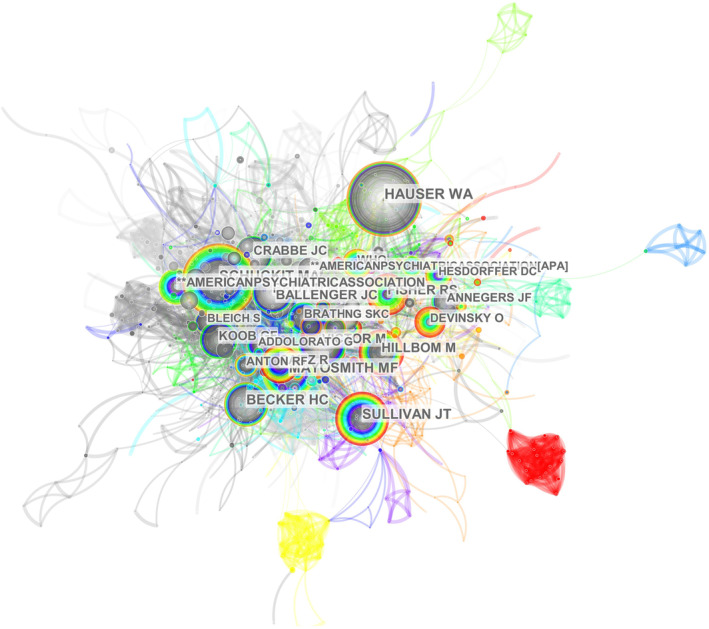
Network visualization of co‐cited authors, where the node represents the author, the size represents the number of articles published, and the node line represents the strength of association.

The top 10 co‐cited authors published more than 81 papers in the field (Table 4). W. Allen Hauser ranked first, with 87 co‐citations, followed by Marc A. Schuckit, John T. Sullivan, Howard C. Becker, and Michael F. Mayo‐Smith. And the co‐cited authors mapping was shown in Figure 6. W. Allen Hauser had the greatest number of co‐citations and collaborated closely with other authors.

### 3.9 Analysis of references

The top five most cited references and their first authors were presented in Table 5. These references were published between 2004 and 2017, and four of five were published after 2014. The most highly cited paper (14 times) was written by Marc A. Schuckit, entitled *Recognition and management of withdrawal delirium* (*delirium tremens*).^27^

**TABLE 5:** Top five references.

Rank	Counts	Reference	First author
1	14	Recognition and management of withdrawal delirium (delirium tremens) [J]	Marc A. Schuckit
2	13	ILAE classification of the epilepsies: position paper of the ILAE Commission for Classification and Terminology [J]	Ingrid E. Scheffer
3	13	Alcohol withdrawal syndrome: mechanisms, manifestations, and management [J]	Stephen Jesse
4	10	Dexmedetomidine as adjunct treatment for severe alcohol withdrawal in the ICU [J]	Samuel G. Rayner
5	10	Management of alcohol withdrawal delirium an evidence‐based practice guideline [J]	Michael F. Mayo‐Smith

### 3.10 Analysis of co‐cited references

**FIGURE 7:**
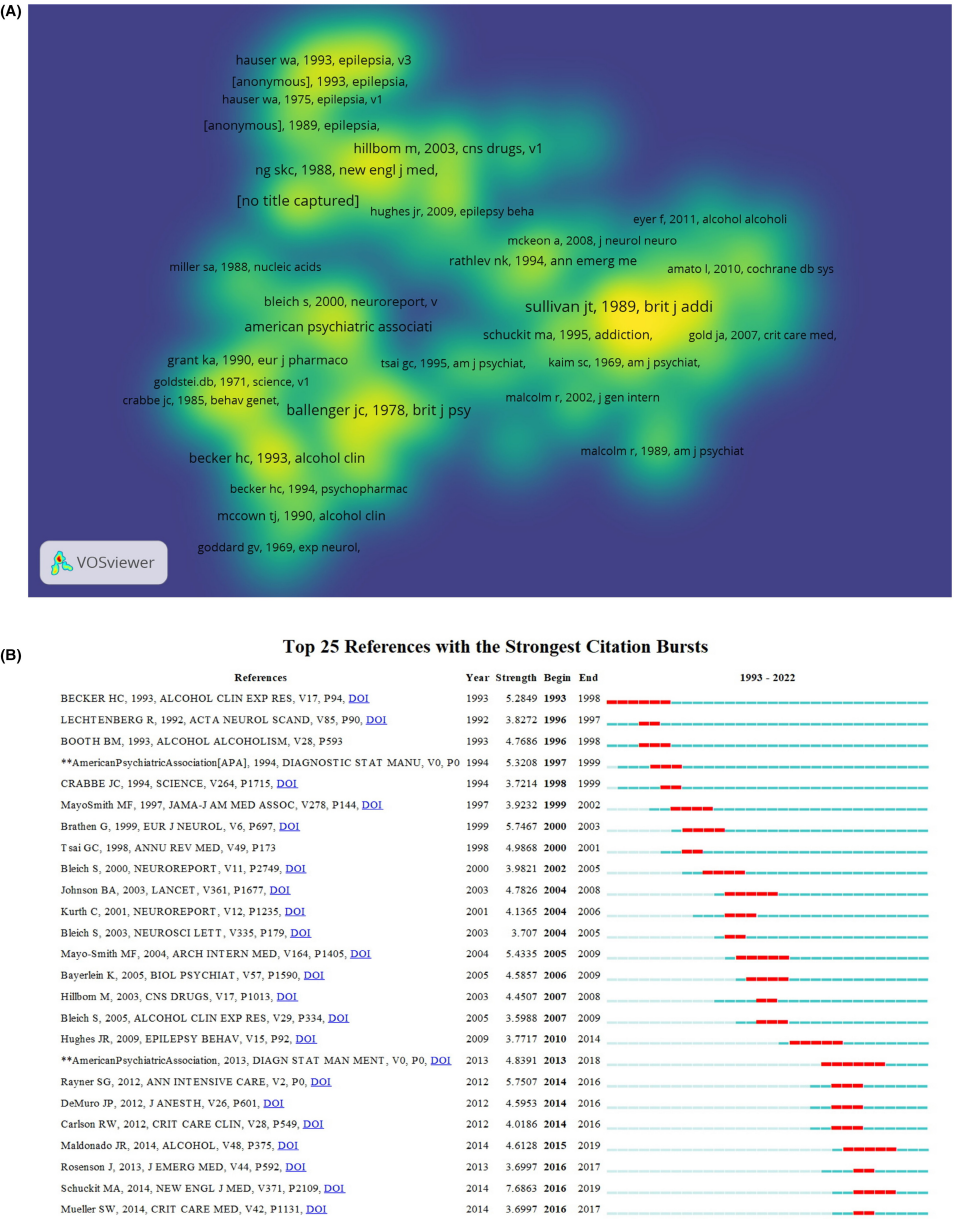
Schematic illustration of analysis of co‐cited references. (A) The density visualization map of co‐cited references based on VOSviewer. The color closer to yellow indicates a higher influence, while blue indicates a lower influence. (B) Burst strength and time duration of the top 25 references with the strongest citation bursts.

Since 1993, there are a total of 79 667 co‐cited references in the field, which were average co‐cited 51 times per article. Among them, Assessment of Alcohol Withdrawal: the revised clinical institute withdrawal assessment for alcohol scale (CIWA‐Ar) had the highest number of citations (*n* = 78) (Figure 7A and Table 6).^28^ In the co‐cited networks, this article was also the most influential research, sharing strong relationships with the rest of the literature. The top 25 co‐cited references with the strongest citation bursts were shown in Figure 7B, and the burst strength of these ranged from 3.59 to 7.65. The strongest ones (Strength = 7.68) came from Marc A. Schuckit, entitled *Recognition and management of withdrawal delirium* (*delirium tremens*).^29^

**TABLE 6:** Top five co‐cited references.

Rank	Co‐cited (counts)	Reference	First author
1	78	Assessment of alcohol withdrawal: the revised clinical institute withdrawal assessment for alcohol scale (CIWA‐Ar) [J]	John T. Sullivan
2	63	Users' guides to the medical literature: XIII. How to use an article on economic analysis of clinical practice B. What are the results and will they help me in caring for my patients? [J]	Bernie J. O'Brien
3	58	Kindling as a model for alcohol withdrawal syndromes [J]	James C. Ballenger
4	55	Alcohol detoxification and withdrawal seizures: clinical support for a kindling hypothesis	Martha E. Brown
5	46	The role of abstinence in the genesis of alcoholic epilepsy [J]	Maurice Victor

### 3.11 Analysis of keywords

**FIGURE 8:**
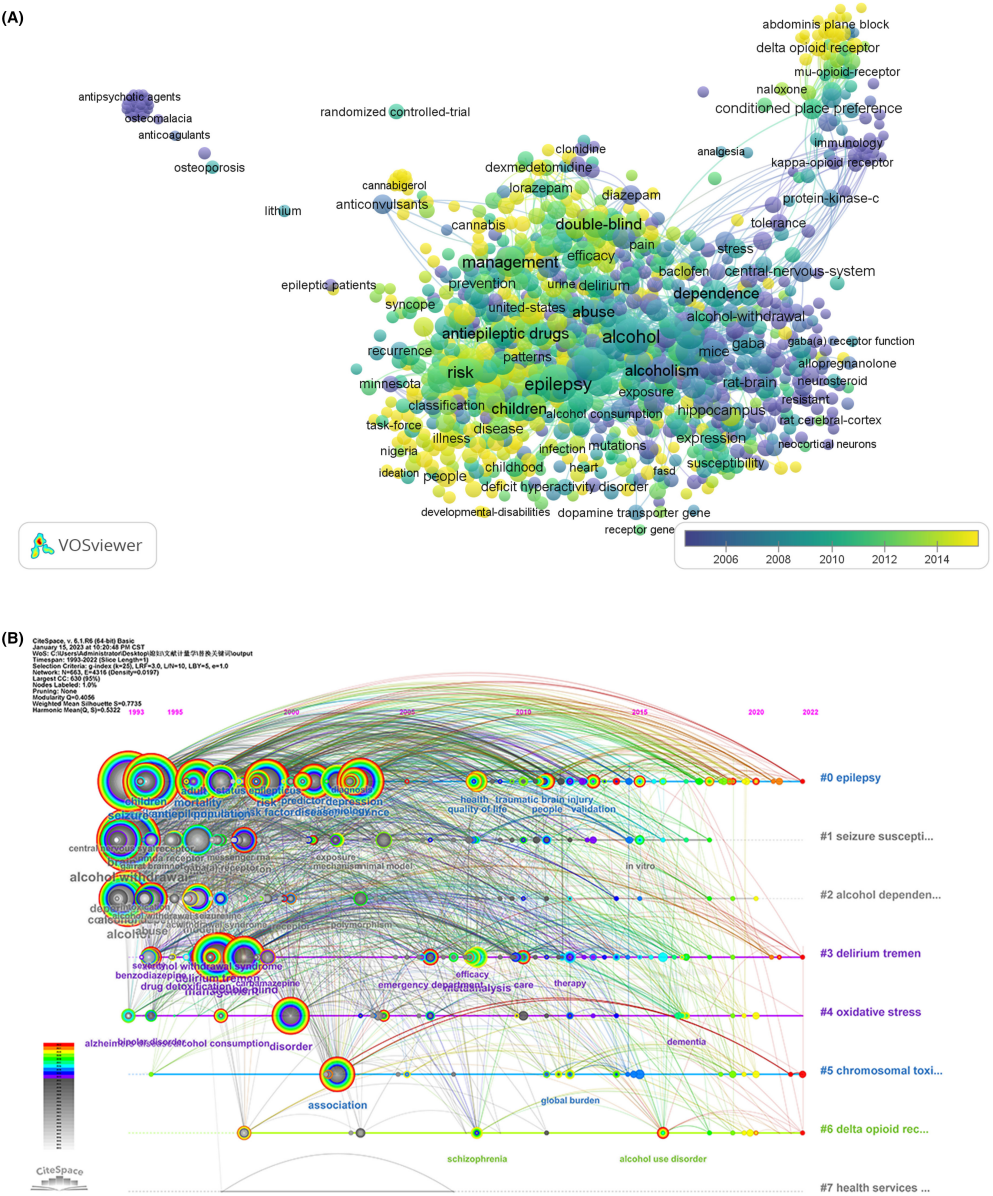
Schematic illustration of analysis of keywords, topics, and themes. (A) The average publication year; (B) Timeline view of the eight clusters. The location of the node indicates the time the keywords first appeared, and the lines indicate the relationships between them.

High‐frequency keywords in articles reflect the hot spots of a research field.^30^ The top five keywords were epilepsy (290, TLS = 1675), alcohol (247, TLS = 1604), seizures (193, TLS = 1203), alcohol withdrawal (113, TLS = 830), and management (104, TLS = 644) (Table 7). The VOSviewer was used to color the keywords according to the average time of occurrence. The keywords, illness, stress, and dopamine transporter, were emerging as a hotspot of fields in recent years (Figure 8A). We also provided the timeline view for the major clusters in Figure 8B. It shows the evolutionary pathway in the study of alcohol and epilepsy. Eight core keywords were arranged as forms of timelines vertically, including epilepsy, seizure susceptibility, alcohol dependence, delirium treatment, oxidative stress, chromosomal toxicity, delta‐opioid receptor, and health services utilization. Each timeline covered a series of keywords.

**TABLE 7:** Top five co‐cited references.

Rank	Keywords	Occurrences	TLS
1	Epilepsy	290	1675
2	Alcohol	247	1604
3	Seizures	193	1203
4	Alcohol withdrawal	113	830
5	Management	104	644
6	Prevalence	101	614
7	Risk	101	595
8	Withdrawal	89	637
9	Epidemiology	85	520
10	Alcoholism	81	560
11	Mortality	77	447
12	Dependence	76	600
13	Double‐blind	71	480
14	Consumption	65	405
15	Brain	65	371
16	Depression	61	423
17	Risk‐factor	60	374
18	Children	60	326
19	Association	54	338
20	Anti‐epileptic drugs	51	297

Abbreviation: TLS, Transport Layer Security.

## 4 DISCUSSION

Alcoholism might be more common among individuals with epilepsy, compared to the general population.^31^, ^32^, ^33^, ^34^ Alcohol, as one of the common cause inducing seizures, has garnered growing interest from researchers. This study quantified scientific productions, countries, institutions, journals, authors, references, and keywords, which may help in identifying the latest progress, evolution paths, frontier research hot spots, and future research trends in this field.

This study covered all available articles until December 31, 2022, from the Web of Science database. The annual growth rate was 16.46% based on the number of published articles in 1993. It presented an increasing trend, reflecting that research in the field was gradually becoming a hot pot.

The types of journals reflect the different research directions.^35^, ^36^ The papers were mainly published in neurology‐related journals. The research was also published in other kinds of journals, including pharmacology, biochemistry, and behavioral journals. The existence of researcher groups in different fields was conducive to interdisciplinary communication.

By analyzing the highly productive authors and co‐cited authors, we found Thomas Sander, Lutz G.Schmidta, and Helmut Harms all belonging to the Free University of Berlin. They focus on the relationships between genes and alcohol‐related seizures.^37^, ^38^, ^39^ Some emerging scholars also pay attention to the field. Yulin Zhao and his collaborators were working on G‐protein‐gated inwardly rectifying K+ (GIRK) channels and seizures. They found that alcohol mitigates the effects of a convulsant in acute epilepsy mouse models by activating GIRK channels.^40^ Yirga Legesse Niriayo and his collaborators revealed that alcohol consumption increased the risk of seizures in epileptic patients.^41^ The emerging scholars were devoted to alcohol and epilepsy, but the literature analysis showed limited cooperation and exchanges between each other. In the future, more cooperation and communication are needed to promote research in the field.

The top 10 productive countries were almost developed, contributing more than 80% of the papers. The USA, with the majority of top institutions, led international collaborations. Studies on alcohol and epilepsy were weaker in Asia, which also has a lot of drinkers. It was indicated that the international distribution of this field was imbalanced. This may be related to the developed economy and high investment in healthcare in developed countries. Therefore, it is necessary to increase the research investment of the Asian countries and cooperation with the developed countries.

The analysis of keywords showed that epilepsy, alcohol, seizures, alcohol withdrawal, and management were the most frequently used keywords in research articles in this area. It is worth mentioning that alcohol withdrawal and management were also the focus of the field. Alcohol withdrawal syndrome occurs when the alcoholics stop drinking suddenly.^42^, ^43^, ^44^, ^45^ Reducing alcohol intake gradually, rather than abruptly, may help prevent alcohol‐withdrawal seizures.^46^ Meanwhile, Downtown Emergency Services Center (DESC) in Seattle, Washington has already implemented alcohol management as a harm reduction strategy. Other countries and institutions may need to pay more attention to alcohol management.

Drinking can induce seizures. Intake of alcohol may make the patients miss the antiepileptic drugs or reduce the efficacy of medications. Surprisingly, small doses of alcohol can help to reduce anxiety and stress, which prevent seizures.^47^, ^48^ Large doses of alcohol may increase γ‐aminobutyric acid (GABA) ergic activity with stimulation of the GABA receptor‐mediated Cl−, which can increase the risk of seizures.^42^, ^49^, ^50^ However, the specific cutoff for alcohol intake remains undetermined. The values of drinking in epileptic patients warrant further studies.

### 4.1 Strengths and limitations

This study also had certain limitations. First of all, our data are only from the Web of Science (SCI & SSCI), which may need to be more comprehensive. In the next place, we screened the English articles, so that articles published in other languages were not included.

## 5 CONCLUSION

This study conducted a quantitative and visual analysis in the field of alcohol and epilepsy. With the help of CiteSpace and VOSviewer, we have a deeper understanding of the latest progress, evolution paths, frontier research hot spots, and future research trends in the field. And the field of epilepsy and alcohol research has received extensive attention from countries and institutions, especially the United States. Critical factors associated with alcohol and epilepsy, such as alcohol withdrawal and management, also become hot spots in the field. The dose of alcohol that epileptics need to control will be the focus of future research. Overall, our results will help researchers understand the research trend, and provide clues for future research directions in the field.

## AUTHOR CONTRIBUTIONS

Wenhui Liu designed and analyzed the data; Wenhui Liu and Huan Li performed the image formation. Wenhui Liu wrote the manuscript. Simei Lin and Huan Li contributed to revising the manuscript, with contributions from all the authors.

## FUNDING INFORMATION

This work was supported by The Second Affiliated Hospital of Guangzhou Medical University Postdoctoral Start‐up Funding: 31010406.

## CONFLICT OF INTEREST STATEMENT

The authors declare no conflict of interest.

## ETHICS STATEMENT

Approval of the Research Protocol by an Institutional Reviewer Board: N/A.

Informed Consent: N/A.

Registry and the Registration No. of the study/trial: N/A.

Animal Studies: N/A.

## Data Availability

Data sharing not applicable to this article as no datasets were generated or analyzed during the current study.
